# Adipose Tissue Macrophages Modulate Obesity-Associated β Cell Adaptations through Secreted miRNA-Containing Extracellular Vesicles

**DOI:** 10.3390/cells10092451

**Published:** 2021-09-17

**Authors:** Hong Gao, Zhenlong Luo, Zhongmou Jin, Yudong Ji, Wei Ying

**Affiliations:** 1 Division of Endocrinology and Metabolism, Department of Medicine, University of California, San Diego, CA 92093, USA; hog007@health.ucsd.edu (H.G.); luozhenlong121@126.com (Z.L.); jiyudong121@126.com (Y.J.); 2 Department of Gastroenterology, Tongji Hospital, Tongji Medical College, Huazhong University of Science and Technology, Wuhan 430030, China; 3 Division of Biological Sciences, University of California, San Diego, CA 92093, USA; zhj048@ucsd.edu; 4 Department of Anesthesiology, Institute of Anesthesiology and Critical Care, Union Hospital, Tongji Medical College, Huazhong University of Science and Technology, Wuhan 430022, China

**Keywords:** adipose tissue macrophage, extracellular vesicle, miRNA, β cell replication, insulin secretion, obesity

## Abstract

Obesity induces an adaptive expansion of β cell mass and insulin secretion abnormality. Expansion of adipose tissue macrophages (ATMs) is a hallmark of obesity. Here, we assessed a novel role of ATMs in mediating obesity-induced β cell adaptation through the release of miRNA-containing extracellular vesicles (EVs). In both in vivo and in vitro experiments, we show that ATM EVs derived from obese mice notably suppress insulin secretion and enhance β cell proliferation. We also observed similar phenotypes from human islets after obese ATM EV treatment. Importantly, depletion of miRNAs blunts the effects of obese ATM EVs, as evidenced by minimal effects of obese DicerKO ATM EVs on β cell responses. miR-155 is a highly enriched miRNA within obese ATM EVs and miR-155 overexpressed in β cells impairs insulin secretion and enhances β cell proliferation. In contrast, knockout of miR-155 attenuates the regulation of obese ATM EVs on β cell responses. We further demonstrate that the miR-155-*Mafb* axis plays a critical role in controlling β cell responses. These studies show a novel mechanism by which ATM-derived EVs act as endocrine vehicles delivering miRNAs and subsequently mediating obesity-associated β cell adaptation and dysfunction.

## 1. Introduction

The prevalence of type 2 diabetes mellitus (T2DM) has risen dramatically in the past couple of decades [1]. Insulin resistance is a central etiologic defect of T2DM [2,3]. Chronic low-grade tissue inflammation, accompanied by an increase in immune cell infiltration, is a hallmark of obesity in both humans and rodents and an important contributor to the pathogenesis of insulin resistance and metabolic diseases [4,5]. Insulin resistance results in a compensatory growth of pancreatic β cells, leading to a status of hyperinsulinemia. Many obese individuals are prediabetic and will eventually develop T2DM characterized by insufficient insulin production.

In the obese state, adipose tissue undergoes significant expansion, concomitant with a state of chronic and unresolved inflammation [4,6,7,8,9,10]. Numerous studies in both humans and rodents have demonstrated that a remarkable accumulation of proinflammatory macrophages is one of the striking components of obesity-induced adipose tissue inflammatory responses [11,12,13,14,15,16]. These proinflammatory macrophages residing in obese adipose tissues (ATMs) are one of the main drivers for the pathogenesis of obesity-induced tissue inflammation and insulin resistance [17,18,19,20,21].

Extracellular vesicles (EVs) and their cargo molecules play roles in many levels of intercellular crosstalk [22,23]. EVs are released into interstitial spaces and blood as well as other body fluids by many cell types in both human and mouse [24,25,26]. Emerging evidence suggests that EV-mediated cellular communications exert profound regulation on metabolic responses in obesity [27,28,29,30,31]. We have shown that obese ATMs can reduce peripheral insulin sensitivity by releasing EV/microRNAs (miRNAs) locally or into the circulation [32]. Among the exosomal miRNAs derived from obese ATMs, we have demonstrated the pathogenic effects of miR-155 on cellular insulin action [32]. In addition, Guay et al. have shown that T cell-derived exosomes containing miR-142 and miR-155 trigger β cell apoptosis, possibly resulting in the development of Type 1 diabetes [33].

Obesity-induced insulin resistance provokes an adaptive expansion of β cell mass, which compensates by secreting increased amount of insulin [34,35,36]. The physiological mechanisms driving obesity-induced β cell expansion include increased plasma glucose concentrations, insulin, expression of hepatocyte growth factors, and islet macrophage-mediated islet inflammation [37,38,39,40,41,42,43]. Furthermore, previous studies have shown the critical roles of key transcription factors or miRNA in regulating the genetic network associated with β cell insulin secretion and proliferation in the context of obesity [44]. For example, a previous study reported that hyperlipidemia-induced miR-155 accumulation facilitates the growth of β cell mass by directly repressing expression of v-maf musculoaponeurotic fibrosarcoma oncogene family, protein B (*Mafb*) [45]. Our previous studies have demonstrated that obese ATMs preferentially secreted miR-155 enriched EVs, leading to an accumulation of this miRNA in the local target cells or at distal sites [32]. However, whether obese ATM-secreted miRNA-containing EVs are capable of regulation of β cell functions in response to obesity remains unknown.

Here, we report that obese ATM-produced EVs can be delivered into β cells, both in vitro and in vivo, resulting in blunted insulin secretion and more proliferating β cells. The ability of obese ATM EVs to mediate β cell responses is mainly dependent on miRNAs, as evidenced by modest effects of obese DicerKO ATM EVs on β cell insulin secretion and replication. We further identify that miR-155 impairs the glucose-stimulated insulin secretion of β cells by suppressing *Mafb* expression. In addition, the miR-155-*Mafb* axis can promote β cell proliferation. In contrast, the depletion of miR-155 partially prevents the effects of obese ATM EVs on β cell responses.

## 2. Materials and Methods

### 2.1. Animals

In the study, 7–8 weeks old C57BL/6 (B6) male mice were fed a high-fat diet (HFD, 60% fat calories, 20% protein calories, and 20% carbohydrate calories; Research Diets) or a normal chow diet ad libitum. MIPGFP (Stock No. 006864), Mki67 (Stock No. 029802), Dicer flox/flox (Stock No. 006366), miR-155 (Stock No. 007745), and LysMCre mice (Stock No. 004781) were purchased from the Jackson Laboratory. To generate myeloid cell-specific Dicer null mice, Dicer flox/flox were bred with transgenic mice harboring Cre recombinase driven by myeloid-specific lysozyme M promoter to create the following genotypes: Dicer flox/flox (control) and LysMCre-Dicer (DicerKO). To generate MIPGFP-Mki67 mice (GFP+RFP+ cells are proliferating ki67+ β cells), MIPGFP (GFP+ cells are β cells) mice were bred with Mki67 (RFP+ cells are proliferating ki67+ cells) mice. All mice were maintained on a 12/12-h light–dark cycle. All animal procedures were performed in accordance with University of California, San Diego Research Guidelines for the Care and Use of Laboratory Animals and all animals were randomly assigned to cohorts when used.

### 2.2. Flow Cytometry Analysis

Unless otherwise specified, we purchased antibodies from Biolegend (San Diego, CA, USA). Stromal cells of epididymal fat were stained with fluorescence-tagged antibodies to macrophages (CD45+CD11b+F4/80+). Data were analyzed using Flowjo software. To measure the population of proliferating β cells, islets were dispersed by 0.02% trypsin-EDTA treatment and then used for the intracellular staining with antibodies against insulin (Cat. No. 565689, BD Biosciences, San Diego, CA, USA), Ki67 (Cat. No. 151204), or pHH3 (Cat. No. 650807).

### 2.3. Isolation of Adipose Tissue Macrophages (ATMs)

Epididymal adipose tissues (VATs) of 16–20 weeks HFD-fed mice were mechanically chopped and then digested with collagenase II (Sigma-Aldrich, St. Louis, MO, USA; Cat. No. C2674) for 15 min at 37 °C. After passing cells through a 200 µm cell strainer (VWR, Cat. No. 100490-158) and centrifugation at 1000× *g* for 10 min, the pellet containing the stromal vascular cell (SVC) fraction was then incubated with red blood cell lysis buffer. SVC single cell suspensions were incubated with fluorescence-tagged antibodies against CD45 (Biolegend, Cat. No. 103116), CD11b (Biolegend, Cat. No. 101206), and F4/80 (Biolegend, Cat. No. 123116). CD45+CD11b+F4/80+ macrophages were purified using SONY MA900 flow cytometer (SONY). In addition, cells were stained with CD11c (Biolegend, Cat. No. 117343) and CD206 (Biolegend, Cat. No. 141715) antibodies to measure the levels of M1 and M2 activation. ATMs were then cultured in IMDM containing 10% exosome-free FBS to produce extracellular vesicles (EVs).

### 2.4. Isolation of Islets

NCD and HFD mice were euthanized, and freshly prepared collagenase P (Roche, Basel, Switzerland; Cat. No. 11249002001) solution (0.5 mg/mL) was injected into the pancreas via the common bile duct. The perfused pancreas was digested at 37 °C for 10 min, and the islets were handpicked under a stereoscopic microscope. To sort out GFP+ or GFP+RFP+ β cells, islets were dispersed into single cell suspensions, and then cells with fluorescent reporters were purified using a SONY MA900 flow cytometer (SONY; San Jose, CA, USA). Human islets (Donor ID: HP-20199-01; BMI = 27.3, HbA1c = 5.1%/32 mmol/mol IFCC, age = 21 years old, COVID-19-negative) were received from Prodo Laboratories (Aliso Viejo, CA, USA).

### 2.5. EV Purification and Pharacterization

The EVs from ATM culture medium/exosome-free FBS were prepared as previously described [32]. After 24-h culture, debris and dead cells in the ATM medium were removed by centrifugation at 1000× *g* for 10 min and then filtrated through a 0.2 µM filter. The medium was then subjected to ultracentrifugation at 100,000× *g* for 4 h at 4 °C. After wash with PBS (100,000× *g* for 20 min), the EV-containing pellet was resuspended in 1 mL PBS and passed through a 0.2 µM filter to remove large particles. The particle size and concentration of ATM EVs were measured by NanoSight analysis (Malvern Instruments, Malvern, UK). To monitor EV trafficking, EVs were labeled with PKH26 fluorescent dye using the PKH26 fluorescent cell linker kit (Sigma-Aldrich, Cat. No. PKH26GL-1KT). After PKH26 staining, the EVs were washed with PBS and collected by ultracentrifugation (100,000× *g* for 2 h) at 4 °C. Finally, PKH26 labeled EVs were resuspended in PBS.

To analyze the characteristics of macrophage-derived EVs by flow cytometer (MA900, SONY), EVs (5 × 10^9^) were incubated with latex beads (1 × 10^8^; ThermoFisher, Waltham, MA, USA, Cat. No. A37304) at 4 °C. After overnight incubation, the beads were washed with PBS twice and finally re-suspended in 200 µL of PBS.

### 2.6. In Vivo and In Vitro EV Treatment

For in vitro glucose-stimulated insulin secretion (GSIS) assays, 0.2 × 10^8^ EVs based on NanoSight analysis were added to 10 islets for 24 h. For in vitro proliferation assays, 50 MIPGFP-Mki67 islets or 200 human islets were treated with 1 × 10^8^ EVs for 72 h. For in vivo treatment, 7 weeks old recipient mice were intravenously injected 1 × 10^9^ EVs twice per week and also fed HFD for 4 weeks.

### 2.7. Argonaut-RNA Co-Immunoprecipitation Assay

After treatment with obese ATM EVs, the Argonaut (Ago) protein immunoprecipitation and purification of total RNAs associated with Ago were performed with the miRNA Target IP Kit (Active Motif, Carlsbad, CA, USA) [32]. miR-155 abundance within these Ago-bound RNAs was measured by qPCR analysis.

### 2.8. Differentiation of Bone Marrow-Derived Macrophages

Bone marrow-derived macrophages (BMDMs) were derived from bone marrow cells isolated from 6–8 weeks old WT male mice as previously described [46].

### 2.9. Depletion of Tissue Macrophages

To deplete tissue macrophages, WT mice fed HFD for 1 week were given clodronate liposomes (80 mg/kg body weight; FormuMax, Sunnyvale, CA, USA, Cat. No. F70101C-N) every 7 days via intraperitoneal injection [15]. The HFD-fed control mice were treated with empty liposomes (FormuMax, Cat. No. F70101-C). After 3 weeks treatment with clodronate liposomes or empty liposomes, islets were isolated.

### 2.10. Transfection of miR-155 Mimics, miR-155 Hairpin Inhibitor, or siRNA

Cy3 labeled miR-155 mimics (2pM/10 islets; 2pM/0.1 × 10^6^ Min6 cells; GE Dharmacon), miR-155 mimics (2pM/10 islets; ThermoFisher Scientific, Cat. No. 4464066), miR-155 hairpin inhibitor (10pM/10 islets; Horizon, Cat. No. IH-310430-08-0002), or siRNA-*Mafb* (10pM/10 islets; Horizon, Cat. No. J-059035-09-0002) were transfected into recipient cells with the lipofectamine RNAiMAX reagent (ThermoFisher Scientific, Waltham, MA, USA, Cat. No. 13778-075). After 24 h, the transfection efficiencies were validated by qPCR analysis.

### 2.11. Co-Culture Assay

After being transfected with Cy3-miR-155 mimics (GE Dharmacon), obese ATMs (0.1 × 10^5^/well) were co-cultured with Min6 cells at a ratio of 1:5 using a Transwell plate (0.4 µM polycarbonate filter, Corning) for 16 h, with Min6 cells placed in the lower chamber and obese ATMs in the upper chamber. After washing with PBS twice, the intensity of Cy3 red fluorescence in Min6 cells was examined by flow cytometer (MA900, SONY, San Jose, CA, USA). To inhibit EV secretion, obese ATMs containing Cy3-miR-155 were pretreated with GW4869 (an inhibitor of neutral sphingomyelinase, 10 µM; Cayman Chemical, Cat. No. 6823-69-4) for 24 h [47]. These obese ATMs were used to co-culture with Min6 cells in the medium containing GW4869 for another 16 h.

### 2.12. Immuno-Fluorescence Staining

Pancreases were cut and snap frozen in optimum cutting temperature (O.C.T., Fisher Healthcare). Six-micrometer cryo-sections of tissue sections were cut and fixed with pre-chilled acetone for 20 min. Immunostaining was performed as previously described [42]. Sections were blocked with 5% normal donkey serum (Jackson ImmunoResearch, West Grove, PA, USA, Cat. No. 017-000-001) before the addition of the insulin antibody (ThermoFisher Scientific, Waltham, MA, USA, Cat. No. 53-9769-80). Nuclei were stained with DAPI (4′,6-Diamidino-2-28 phenylindole dihydrochloride). After washing, slides were incubated with Antifade Mounting Medium (VECTASHIELD, Burlingame, CA, USA, Cat. No. ZF0612) and covered with coverslip. Images were acquired on Keyence Fluorescent Microscope and were processed with ImageJ (NIH). The ratio (%) of islet area was calculated by normalizing each area of insulin+ cell area by the total area of the pancreas as previously described [48,49].

### 2.13. Quantitative Reverse Transcriptase-Polymerase Chain Reaction (RT-PCR) Analysis

Total RNA was extracted from either islets or GFP+ β using the RNA extraction protocol according to the manufacturer’s instructions (Zymo Research, Irvine, CA, USA, Cat. No. R1051). cDNA was synthesized using SuperScript III and random hexamers. qPCR was carried out in 10 μL reactions using iTaq SYBR Green supermix on a StepOnePlus Real-Time PCR Systems (ThermoFisher Scientific, Waltham, MA, USA,). The data presented correspond to the mean of 2^−ΔΔCt^ from at least three independent experiments after being normalized to β-actin.

### 2.14. Glucose-Stimulated Insulin Secretion Assays

Primary mouse islets, human islets, or Min6 cells were used to evaluate the effects of macrophage EVs, miR-155, or *Mafb* knockdown on GSIS. Static GSIS experiments were conducted as described previously [42]. Briefly, after washing twice with 1 g/L glucose DMEM, islets were incubated overnight in 1 g/L glucose DMEM, at 37 °C, 5% CO_2_. The following day, islets were washed with fresh 2.8 mM glucose Krebs Ringer Bicarbonate buffer (KRB buffer; 2.6 mM CaCl_2_/2H_2_O, 1.2 mM MgSO_4_/7H_2_O, 1.2 mM KH_2_PO_4_, 4.9 mM KCl, 98.5 mM NaCl, and 25.9 mM NaHCO_3_, supplemented with 20 mM HEPES and 0.2% BSA), and then fasted with 2.8 mM glucose KRB buffer for 30 min. Islets were then incubated for 60 min in 2.8 mM or 16.3 mM glucose KRB buffer. Insulin concentrations in the supernatant were determined using insulin ELISA kits (Alpco, Salem, NH, USA, Cat. No. 80-INSHU-E01.1; Cat. No. 80-INSHU-E01.1). To calculate the relative insulin secretion for each group, the insulin levels of 2.8 mM glucose treatment were used as the basal insulin, and 16.3 mM glucose-stimulated insulin levels were normalized to the basal insulin. Alternatively, the glucose-stimulated insulin secretion data were normalized to total insulin content of the islets or Min6 cells. In another set of islets or Min6 cells, after treatment with ATM EVs, miR-155 mimics, or siRNA-*Mafb*, total insulin content was normalized to total protein of these cells.

### 2.15. Statistics

Tests used for statistical analyses are described in the figure legends. All experiments were repeated at least twice independently. To assess whether the means of two groups are statistically different from each other, an unpaired two-tailed Student’s *t* test was used for statistical analyses using Prism8 software (GraphPad software v8.0; Prism, La Jolla, CA, USA). *p* values of 0.05 or less were statistically significant. Degrees of significance were indicated in the figure legends. For the results of glucose and insulin tolerance tests, statistical comparisons between two groups at each time point were performed with unpaired two-tailed Student’s *t* test, one- or two-way ANOVA.

## 3. Results

### 3.1. Adipose Tissue Macrophage-Produced EVs Are Pathogenic for Insulin Secretion in Obesity

**Figure 1:**
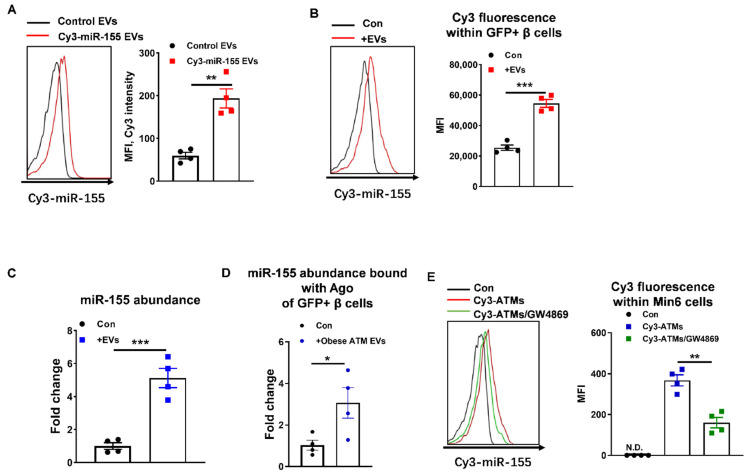
Obese adipose tissue macrophages (ATMs) secrete extracellular vesicles (EVs) as cargos delivering miRNAs into β cells. (**A**) The delivery of Cy3-miR-155 mimics into EVs derived from obese ATMs transfected with Cy3-miR-155 mimics by flow cytometric analysis. (**B**) Cy3 red fluorescent intensity within GFP+ β cells after MIPGFP islets co-cultured with Cy3-miR-155 containing obese ATM EVs by flow cytometric analysis. (**C**) miR-155 abundance in islets co-cultured with obese ATM EVs. (**D**) The abundance of miR-155 bound with argonaut (Ago) proteins of GFP+ β cells after treatment of MIPGFP islets with obese ATM EVs. (**E**) Cy3 red fluorescent intensity within Min6 cells co-culutred with Cy3-miR-155 containing obese ATMs in a transwell plate with EV secretion inhibitor GW4869 (10 µM) by flow cytometric analysis. Data are presented as the mean ± SEM. * *p* < 0.05, ** *p* < 0.01, *** *p* < 0.001, Student’s *t* test (**A**–**D**) or one-way ANOVA (**E**).

We first examined if ATMs (F4/80+CD11b+) can release EVs harboring miRNAs that are then delivered into β cells. After transfection of red fluorescent Cy3-labeled miR-155 mimics, obese ATMs secreted EVs containing Cy3-miR-155 mimics, as shown by the presence of a robust Cy3 fluorescence in these obese ATM EVs (Figure 1A). In addition, we have shown that ATM-derived miRNA-containing EVs can be taken up into β cells, as evidenced by detection of Cy3-miR-155 in GFP+ β cells (Figure 1B). Concomitantly, miR-155 was highly enriched in these recipient cells cocultured with obese ATM EVs (Figure 1C). Additionally, more miR-155 molecules were bound with Ago proteins of GFP+ β cells after treatment of NCD MIPGFP islets with obese ATM EVs (Figure 1D). Furthermore, prior treatment of obese ATMs with an EV synthesis inhibitor GW4869 remarkably reduced EV production and the delivery of Cy3-miR-155 from ATMs into Min6 cells in a transwell (pore size = 0.4 µm) coculture, demonstrating that EVs are important vehicles extracellularly transporting miRNAs (Figure 1E). Overall, our results show that ATM-derived miRNA-containing EVs can be taken up into pancreatic β cells.

**Figure 2:**
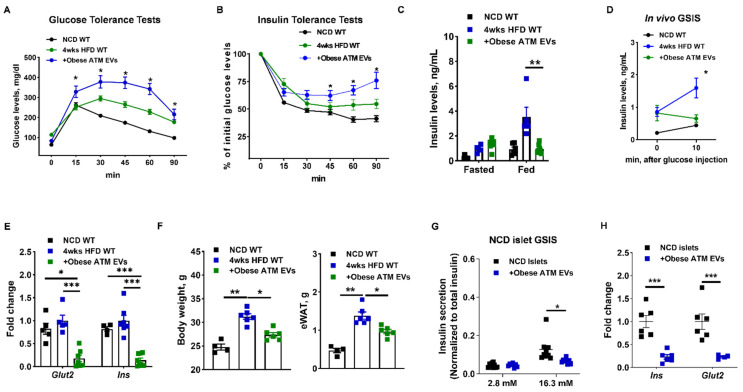
Obese ATM EVs blunt the insulin secretion of obese mice. (**A**,**B**) Glucose and insulin tolerance tests were performed in high fat diet (HFD)-fed WT recipient mice after 4 weeks obese ATM EV treatment. (**C**) Insulin levels after 16 h fasting or feeding. (**D**) The insulin levels of HFD recipient mice after 10 min glucose injection. (**E**) The abundance of genes associated with insulin secretion in the islets of HFD recipients. (**F**) The epididymal fat mass and body weight of HFD mice after 4 weeks obese ATM EV treatment. (**G**) The effect of obese ATM EVs on the glucose-stimulated insulin secretion (GSIS) of islets isolated from normal chow diet (NCD) fed mice. (**H**) The expression of *Ins* and *Glut2* in NCD islets treated with obese ATM EVs. Data are presented as the mean ± SEM. A–B, D, N = 5–8 per group. * *p* < 0.05, ** *p* < 0.01, *** *p* < 0.001, Student’s *t* test (**C**,**D**,**G**,**H**), one-way (**E**,**F**) or two-way (**A**,**B**) ANOVA.

Adipose tissue macrophages play critical roles in the pathogenesis of tissue inflammation and insulin resistance in obesity [4]. Our previous studies have demonstrated the critical role of ATM-derived EV/miRNAs in mediating peripheral insulin sensitivity [32]. Thus, to examine the in vivo pathogenic effects of obese ATM EV/miRNAs on β cell functions, obese ATM EVs (1 × 10^9^ EVs/mouse; Figure S1A) were intravenously injected into a group of obese WT mice. Concordant with an efficient delivery into Min6 cells or GFP+ β cells in vitro, obese ATM EVs can be readily transported into pancreatic β cells, as evidenced by the appearance of a robust red fluorescence in the pancreatic insulin-producing cells of recipients after an injection of PKH26-labeled obese ATM EVs (Figure S1B). To determine the roles of ATM EVs in regulating β cell responses during the development of obesity, WT recipients were intravenously injected with obese ATM EVs (1 × 10^9^ EVs/mouse, twice injection per week) and also started feeding HFD. After 4 weeks, all HFD-fed mice had higher population of ATMs and proinflammatory M1-like (F4/80+CD11b+CD206-CD11c+) macrophages than in lean WT mice (Figure S1C). There was no difference in ATM population and activation among all HFD-fed mice (Figure S1C). In line with our previous findings, the recipients injected with obese ATM EVs exhibited impaired glucose tolerance and insulin sensitivity (Figure 2A,B). Compared to the controls, obese ATM EV treatment resulted in significantly lower insulin levels in the fed state (Figure 2C), concomitant with higher glucose levels (Figure S2A). In addition, obese ATM EVs-treated mice failed to produce additional insulin after glucose injection, whereas the control HFD-fed mice elevated insulin secretion in response to glucose stimulation (Figure 2D). Consistently, islets isolated from obese ATM EVs-treated recipients had notably lower expression levels of genes associated with insulin secretion than the control islets (Figure 2E). Concomitant with obese ATM EVs-induced reduction in circulating insulin, obese-ATM EVs treated mice had blunted adipose tissue expansion after 4 weeks HFD feeding and lower body weight (Figure 2F). qPCR analysis also indicated that obese ATM EV treatment resulted in a significant repression on the expression of genes associated with lipogenesis but not lipolysis (Figure S2B). Obese ATM EVs (0.2 × 10^8^ EVs/10 islets) also blunted glucose-stimulated insulin secretion (GSIS) of NCD WT islets in vitro (Figure 2G,H), further confirming the suppressive effect of obese ATM EVs on β cell insulin secretion. We also found a reduction in intracellular insulin content after treatment of NCD WT islets with obese ATM EVs (Figure S2C). However, there were non-significant effects of obese ATM EVs on the GSIS and intracellular insulin content of islets isolated from 12 weeks HFD WT mice (Figure S2D,E). Overall, these results suggest that obese ATMs can impair the insulin secretion of β cells through releasing pathogenic EVs.

### 3.2. Obese ATM-Derived EVs Can Promote Obesity-Induced β Cell Proliferation

**Figure 3:**
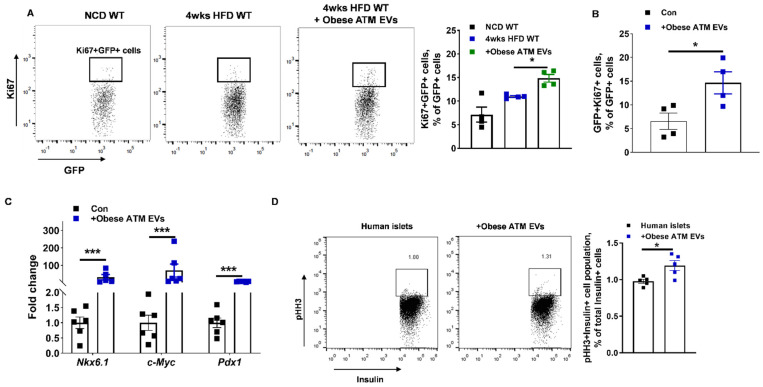
Obese ATM EVs enhance β cell proliferation in response to obesity. (**A**) The population of Ki67+GFP+ cells in the islets isolated from NCD MIPGFP-Mki67 WT, 4 weeks HFD MIPGFP-Mki67 WT, or 4 weeks HFD MIPGFP-Mki67 WT mice treated with obese ATM EVs. (**B**) The in vitro effect of obese ATM EVs on the Ki67+GFP+ cell proportion of NCD MIPGFP-Mki67 islets. (**C**) The abundance of genes associated with β cells proliferation after 72 h obese ATM EV treatment. (**D**) Effect of obese ATM EVs on the population of pHH3+Insulin+ cells in human islets. Data are presented as the mean ± SEM. N = 3–4 (**A**,**B**), N = 6 (**C**), N = 5 (**D**) per group. * *p* < 0.05, *** *p* < 0.001, Student’s *t* test (**B**–**D**) or one-way (**A**) ANOVA.

Obesity-induced insulin resistance results in a compensatory expansion of pancreatic β cell mass. To examine the mechanism by which ATM-derived EVs enhance β cell proliferation, obese ATM EVs (1 × 10^9^ EVs/mouse, twice injection per week) were intravenously injected into a group of HFD-fed MIPGFP-Mki67 mice which have fluorescently labelled proliferating ki67+ β cells (GFP+RFP+). After 4 weeks of HFD feeding, the control MIPGFP-Mki67 mice had a modest increase in the proportion of proliferating ki67+ β cells compared to the lean MIPGFP-Mki67 mice (Figure 3A). Notably, 4 weeks HFD-fed MIPGFP-Mki67 mice injected with obese ATM EVs displayed a significant enhancement in proliferating ki67+ β cell proportion, compared to the control obese MIPGFP-Mki67 mice (Figure 3A). Consistently, obese ATM EV treatment led to increased levels of GFP+ β cells and islet area in these mice, but in the obese ATM EV-treated mice, flow cytometric analysis indicates less insulin intensity after normalized to GFP+ cell population (Figure S3A,B). We also observed an increase in β cell proliferation 72 h after treatment of NCD MIPGFP-Mki67 islets with obese ATM EVs (1 × 10^8^ EVs/50 islets) (Figure 3B). qPCR analysis also confirmed that obese ATM EV treatment increased expression of genes associated with β cell mass expansion (Figure 3C). In addition, there was no difference in apoptosis between controls and the islets treated with obese ATM EVs (Figure S3C). We also found that obese ATM EV treatment increased the population of proliferating Insulin+ cells in human islets, as shown by increased proportion of ki67+Insulin+ cells or pHH3+Insulin+ cells (Figure 3D and Figure S3D–F). Thus, these data indicate that obese ATM EVs elevated β cell proliferation during early stage of obesity.

### 3.3. miRNAs Are Indispensable Components for the Regulation of Obese ATM-Derived EVs on β Cell Responses

**Figure 4:**
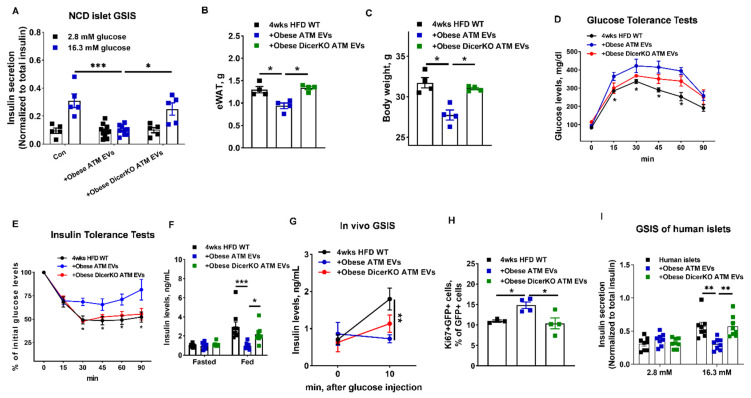
The importance of miRNAs in obese ATM EVs. (**A**) The GSIS of NCD islets after either obese ATM EV or obese DicerKO ATM EV treatment. After 4 weeks obese ATM EV or obese DicerKO ATM EV treatment, the epididymal fat mass (**B**), body weight (**C**), glucose and insulin tolerance tests (**D**,**E**), insulin levels after 16 h fasting or feeding (**F**), and glucose-stimulated insulin levels (**G**) of HFD recipients were measured. (**H**) The effect of obese ATM EVs on Ki67+GFP+ cell population of 4 weeks HFD MIPGFP-Mki67 mice. (**I**) The GSIS of human islets after either obese ATM EV or obese DicerKO ATM EV treatment. Data are presented as the mean ± SEM. (**D**,**E**,**G**), N = 5 per group. * *p* < 0.05, ** *p* < 0.01, *** *p* < 0.001, one-way (**B**,**C**,**G**,**H**) or two-way (**A**,**D**,**E**,**F**,**I**) ANOVA.

To demonstrate that obese ATM EVs-induced β cell responses are dependent on miRNA functions, we collected miRNA-free EVs from obese ATMs without Dicer, an essential ribonuclease for the production of mature miRNAs (Figure S4A) [50]. The absence of miRNAs in obese DicerKO ATMs was confirmed by qPCR analysis of miR-155 that is highly expressed in obese ATMs (Figure S4B). We observed that obese DicerKO ATMs produced similar amount of EVs with obese WT ATMs, and there was no difference in the abundance of EV-associated markers (Figure S4C,D). Consistently, treatment of β cells with obese ATM EVs led to impaired GSIS, whereas WT islets treated with miRNA-free EVs had comparable levels of insulin secretion after glucose stimulation with the control cells (Figure 4A). Additionally, mice treated with miRNA-free EVs had comparable metabolic phenotypes with the controls, as measured by fat mass, body weight, GTT, ITT, insulin levels, and GSIS (Figure 4B–G). Similarly, HFD-fed MIPGFP-Mki67 mice treated with miRNA-free EVs had a comparable population of GFP+RFP+ proliferating β cells with the control HFD-fed WT mice (Figure 4H). While obese ATM EV treatment decreased insulin release and intracellular insulin content of human islets, miRNA-free EV treatment had minimal effects on the insulin secretion of human islets (Figure 4I and Figure S4E). These results indicate that miRNAs are important components which contribute to the ability of obese ATM EVs to regulate peripheral insulin sensitivity and β cell functions.

### 3.4. miR-155 Is a Functional Molecule within Obese ATM EVs Mediating β Cell Responses

**Figure 5:**
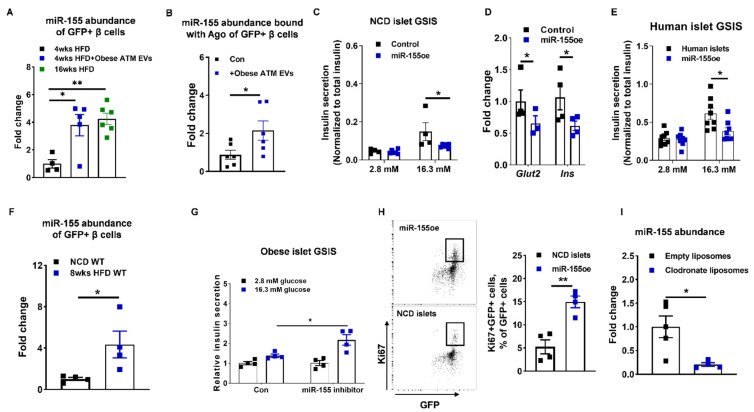
miR-155 accumulation leads to a reduction in insulin secretion but an increase in β cell proliferation. (**A**) miR-155 abundance of GFP+ cells after 4 weeks HFD MIPGFP-Mki67 mice treated with obese ATM EVs for 4 weeks. (**B**,**C**) The effect of miR-155 overexpression (miR-155oe) on the GSIS and genes associated with insulin secretion of NCD islets. (**E**) Effect of miR-155 overexpression on human islet GSIS. (**F**) miR-155 abundance of GFP+ cells isolated from NCD or 8 weeks HFD MIPGFP-Mki67 mice. (**G**) The GSIS of obese islets after transfection of miR-155 inhibitor. (**H**) The proportion of Ki67+GFP+ cells in NCD MIPGFP-Mki67 islets transfected with miR-155 mimics. (**I**) miR-155 abundance in islets of obese mice treated with either empty liposomes or clodronate liposomes. Data are presented as the mean ± SEM. N = 4 (**A**,**C**,**D**,**F**–**H**), N = 5–6 (**B**,**I**), N = 8 (**E**) per group. * *p* < 0.05, ** *p* < 0.01, Student’s *t* test (**B**,**D**,**F**,**H**,**I**), one-way (**A**) or two-way (**C**,**E**,**G**) ANOVA.

Given the critical roles of miRNAs in obese ATM EVs, we next explored the mechanisms by which obese ATM-derived EV miRNAs regulate β cell responses. The majority of obese ATMs are proinflammatory macrophages that preferentially produce miR-155 (Figure S5A), and our previous studies established that miR-155 is a highly enriched miRNA in obese ATM EVs [32,51]. We now show that 4 weeks of obese ATM EV treatment resulted in significant miR-155 accumulation in recipient GFP+ β cells compared to the controls (Figure 5A). In addition, miR-155 abundance in GFP+ β cells isolated from 4 weeks HFD mice treated with obese ATM EVs were similar to that in 16 weeks HFD WT mice (Figure 5A). We also found that there was increased abundance of miR-155 bound with Ago protein of GFP+ cells in islets of recipient MIPGFP mice after 4 weeks of obese ATM EV treatment (Figure 5B). More miR-155 accumulated in human islets after treatment with obese ATM EVs (Figure S5B). We next examined the impact of miR-155 on insulin secretion by overexpression of miR-155 in β cells. The lean islets transfected with miR-155 mimics showed a remarkable decrease in insulin secretion in the presence of high glucose concentration (16.3 mM) (Figure 5C and Figure S5C). The expression of genes associated with insulin secretion were also significantly reduced in the GFP+ β cells with miR-155 overexpression compared to the controls (Figure 5D and Figure S5D). We also found that human islets transfected with miR-155 mimics contained lower level of insulin and secreted less insulin in response to glucose stimulation, compared to the control cells (Figure 5E and Figure S5E,F). Transfection of miR-155 mimics also blunted insulin production and insulin secretion in Min6 cells (Figure S5G,H). Interestingly, obesity resulted in miR-155 accumulation in β cells, as evidenced by a higher miR-155 abundance in the GFP+ β cells isolated from 8 weeks HFD MIPGFP mice than in the β cells of lean MIPGFP mice (Figure 5F). Four weeks HFD feeding led to a modest increase in β cells miR-155 abundance (Figure S5I). Notably, repression of miR-155 activity by transfection of a miRNA hairpin inhibitor can restore the GSIS of obese islets (Figure 5G). In contrast to the suppressive effect on insulin secretion, miR-155 overexpression resulted in a higher percentage of proliferating ki67+ β cells (GFP+RFP+) in islets isolated from NCD MIPGFP-Mki67 mice (Figure 5H). Thus, these data suggest that accumulation of miR-155 in β cells has a dual impact on β cells by impairing insulin secretion and promoting β cell proliferation.

Obesity switches macrophage polarization from a predominantly anti-inflammatory state to proinflammatory state, resulting in increased secretion of miR-155 [32]. Thus, we next determined the contribution of macrophages to miR-155 abundance in target cells by depletion of macrophages using clodronate liposome (Figure S5J). Notably, depletion of macrophages in obese WT mice resulted in less miR-155 abundance in islets, compared to the obese control treated with empty liposomes (Figure 5I). In addition, to examine the regulation of hyperglycemia or hyperlipidemia on islet miR-155 abundance, islets isolated from lean WT mice were treated with glucose (16.3 mM) or palmitate acid (500 µM) in vitro. After 24 h treatment, miR-155 expression was remarkably suppressed in these islets, compared to the control cells (Figure S5K). Therefore, these data suggest that macrophages are the major contributors to obesity-induced accumulation of miR-155 in islets.

**Figure 6:**
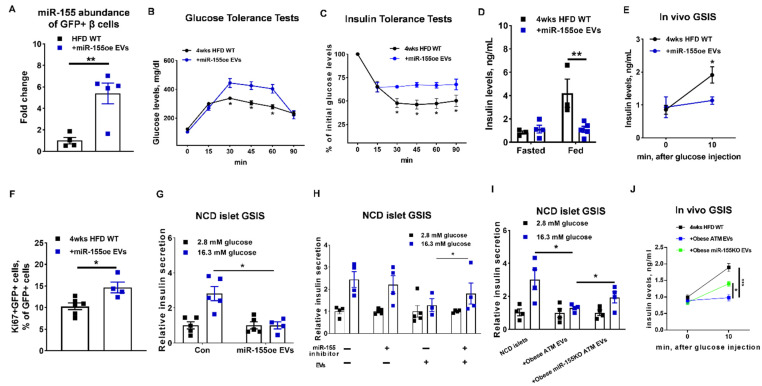
miR-155 is a key molecule contributing to the ability of obese ATM EVs to regulate β cell responses. After 4 weeks treatment of miR-155-enriched BMDM EVs (miR-155oe EVs), the miR-155 expression of GFP+ cells (**A**), glucose and insulin tolerance tests (**B**,**C**), insulin levels after 16 h fasting or feeding (**D**), and glucose-induced insulin levels (**E**) of HFD MIPGFP-Mki67 recipients were measured. (**F**) The population of ki67+GFP+ cells in the islets of HFD MIPGFP-Mki67 mice after 4 weeks treatment of miR-155oe EVs. (**G**) The effect of miR-155oe EVs on NCD islet GSIS. (**H**) The GSIS of NCD islets treated with miR-155 inhibitor or obese ATM EVs. (**I**) The GSIS of NCD islets treated with EVs derived from either obese ATMs or obese miR-155KO ATMs. (**J**) Effect of miR-155 depletion on the ability of obese ATM EVs to regulate GSIS of HFD-fed mice. For (**B**–**G**), control islets or mice were treated with DicerKO BMDM EVs. Data are presented as the mean ± SEM. (**B**,**C**,**E**,**J**), N = 5 per group; (**G**–**I**), N = 4 per group. * *p* < 0.05, ** *p* < 0.01, *** *p* < 0.001, Student’s *t* test (**A**,**E**,**F**), one-way (**J**) or two-way (**B**–**D**,**G**–**I**) ANOVA.

To further validate that miR-155 is one of the functional miRNAs contributing the ability of obese ATM EVs to mediate β cell functions, we collected the miR-155 enriched EVs from the DicerKO bone marrow-derived macrophages (BMDMs; lack of mature miRNAs) transfected with miR-155 mimics (Figure S6A). We confirmed that the transfected miR-155 mimics can be packed into BMDM-derived EVs, as evidenced by a strong Cy3 red fluorescence detected in the EVs secreted from the BMDMs overexpressed with Cy3-miR-155 (Figure S6B). In addition, we observed the appearance of Cy3 red fluorescence in pancreas after intravenous injection of Cy3-miR-155 enriched BMDM EVs (Figure S6C). qPCR analysis also indicated an efficient delivery of miR-155 into GFP+ β cells after treatment of HFD-fed MIPGFP-Mki67 mice with miR-155-enriched BMDM EVs for 4 weeks (Figure 6A). Compared to the control mice, 4 weeks HFD-fed MIPGFP-Mki67 recipients showed impaired glucose tolerance, insulin sensitivity, insulin levels, and in vivo GSIS after 4 weeks injection of miR-155-enriched BMDM EVs (Figure 6B–E). These mice treated with miR-155-enriched BMDM EVs had a significant reduction in fat mass and body weight, compared to the control mice (Figure S6D,E). Additionally, there was an increased population of proliferating ki67+ β cells (GFP+RFP+) after 4 weeks miR-155-enriched BMDM EV treatment (Figure 6F). Consistent with the suppressive effect of miR-155-enriched macrophage EVs on in vivo GSIS, miR-155-enriched BMDM EV treatment resulted in decreased glucose-stimulated insulin secretion, compared to the control cells (Figure 6G).

We next evaluated the impact of miR-155 depletion on the ability of obese ATM EVs to blunt insulin secretion. Treatment of miR-155 inhibitor partially restored the GSIS of islets exposed to obese ATM EVs (Figure 6H). We also collected the miR-155 free EVs from obese miR-155KO ATMs. Depletion of miR-155 from ATM EVs alleviated the pathogenic effect of obese ATM EVs on insulin secretion, as evidenced by a greater GSIS of islets or mice treated with miR-155-free EVs than those treated with obese ATM EVs (Figure 6I,J). However, after glucose stimulation, miR-155 free EV-treated islets produced less insulin than the control cells, suggesting that miR-155 is likely not the only pathogenic molecule in obese ATM EVs (Figure 6I,J). Taken together, these results demonstrate that miR-155 is one of the key functional molecules contributing to the ability of obese ATM EVs to regulate β cell functions.

### 3.5. The miR-155-Mafb Axis Exerts Profound Regulation on the Insulin Secretion and Proliferation of β Cells

**Figure 7:**
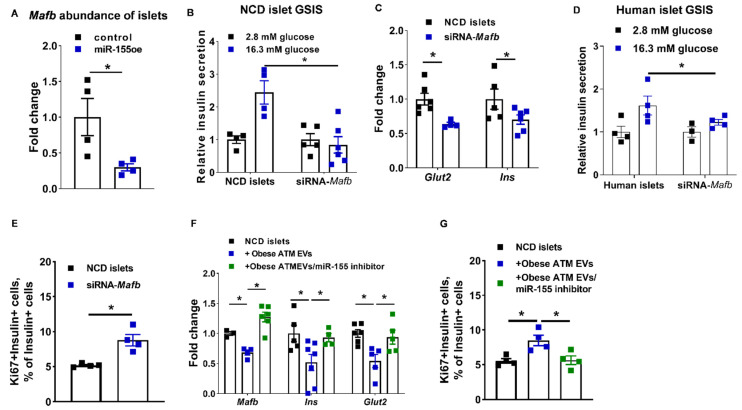
The miR-155-*Mafb* axis exerts profound regulation on β cell functions. (**A**) The *Mafb* abundance of islets transfected with miR-155 mimics. (**B**,**C**) The GSIS and expression of *Glut2* and *Ins* after transfection of siRNA-*Mafb* into NCD islets. (**D**) Effect of *Mafb* knockdown on human islet GSIS. (**E**) The population of Ki67+GFP+ cells in the NCD MIPGFP-Mki67 islets transfected with siRNA-*Mafb*. (**F**,**G**) The effects of miR-155 inhibitor on *Mafb*, *Ins*, and *Glut2* abundance and β cell proliferation of NCD MIPGFP-Mki67 islets treated with obese ATM EVs. N = 4 (**A**,**D**,**E**,**G**), N = 4–6 (**B**,**F**) per group. Data are presented as the mean ± SEM. * *p* < 0.05, Student’s *t* test (**A**,**C**,**E**,**F**), one-way (**G**) or two-way (**B**,**D**) ANOVA.

It has been demonstrated that miR-155 regulates β cell functions by suppressing the expression of *Mafb* [45]. Consistent with these previous reports, miR-155 accumulation in β cells was accompanied by a significant decrease in *Mafb* abundance (Figure 7A and Figure S7A,B). We also found that obese ATM EV treatment or transfection of miR-155 mimics led to decreased *Mafb* expression in human islets (Figure S7C). We further confirmed the critical role of *Mafb* in facilitating β cell insulin synthesis and secretion, as shown by a significant reduction in intracellular insulin abundance and GSIS of both human and mouse islets after a short interfering RNA-induced *Mafb* downregulation (Figure 7B–D and Figure S7C–G). Transfection of siRNA-*Mafb* also resulted in decreased insulin production and secretion in Min6 cells (Figure S7H,I). By contrast, compared to the control MIPGFP-Mki67 islets, knockdown of *Mafb* led to an increase in the population of GFP+RFP+ proliferating ki67+ β cells (Figure 7E). By contrast, suppression of miR-155 function by transfection of a miRNA hairpin inhibitor can restore the expression level of *Mafb* in β cells treated with either obese ATM EVs, accompanied by the recovery of GSIS and β cell proliferation (Figure 6H and Figure 7F,G). Thus, these results suggest that the miR-155 regulates β cell responses through a direct inhibition of *Mafb*.

## 4. Discussion

Here, we present a novel mechanism by which miRNA-containing EVs derived from obese ATMs exert profound regulation on insulin secretion and β cell proliferation. Both in vivo and in vitro experiments confirm that ATM-derived EVs from obese mice are delivered into β cells and result in blunting of insulin secretion and enhanced β cell proliferation. miRNAs are the key components within obese ATM EVs, as evidenced by modest effects of obese DicerKO ATM EVs on β cell functions. Among the identified miRNAs within obese ATM EVs, a high abundance of miR-155 contributes to the capacity of obese ATM EVs to regulate β cell functions through directly repressing on *Mafb* expression. Taken together, these findings highlight a novel role of adipose tissue macrophages in modulating β cell adaptive proliferation and insulin secretion dysfunction in obesity.

Emerging evidence, including our previous studies, indicates that EVs serve as important vehicles to transfer a variety of signals between adjacent or distant cells [23,32,33,52]. In the current study, we have shown that obese ATMs released extracellular miRNAs into β cells, as demonstrated by a robust enrichment of Cy3-miR-155 in β cells after either cocultured with the obese ATMs harboring Cy3-miR-155 in a transwell system or an addition of obese ATM EVs encapsulating Cy3-miR-155. Supplementation of EV secretion inhibitor GW4869 in the transwell coculture system significantly reduced the amount of Cy3-miR-155 accumulation in Min6 cells, thus demonstrating the critical role of EVs as carriers for obese ATMs-derived extracellular miRNAs. Concordantly, our in vivo studies also confirmed the efficient delivery of miRNA-containing obese ATM EVs into pancreatic β cells after an intravenous injection of Cy3-miR-155 containing EVs or PKH26-labeled EVs into WT recipient mice.

The abundance of adipose tissue macrophage population dramatically increases in obesity following the expansion of adipose tissues [11]. By contrast, mouse islet macrophages population remains stable during the early stage of HFD feeding (<16 weeks) [42]. In addition, we observed no difference in miR-155 abundance between lean islet macrophages and obese (20 weeks HFD feeding) islet macrophages (Figure S5L). A major piece of evidence for the new role of obese ATM EVs in regulating β cell functions comes from the reconstitution of obese ATMs-secreted EVs in vivo by intravenous injection into the recipients starting HFD feeding. Additionally, this mouse model can avoid the impact of high levels of endogenous ATM EVs and miR-155 accumulation in β cells in prolonged obese mice. Indeed, we found that obese ATM EV treatment had minimal effects on the GSIS and intracellular insulin content of obese islets (Figure S1G,H). In line with previous findings [14,53], 4 weeks of HFD feeding led to modest growth rates of β cell mass and proinflammatory M1 ATM population. However, multiple intravenous injections of obese ATM EVs to HFD recipient mice impaired glucose-stimulated insulin secretion and insulin levels in the fed state and enhanced proliferation of ki67+ β cells. Obese ATM EVs treated mice had lower adipose tissue mass and body weight after 4 weeks HFD feeding likely due to the lower circulating insulin and thus less adipogenesis. Given the magnitude of the effects of obese ATM EVs in vivo, we also confirmed the regulation of these EVs on islet functions in cell based in vitro experiments. Previous studies have observed that obesity induces proliferation of β cell mass but does not alter the capacity of insulin secretion [54], possibly due to these dual effects of ATM EVs on β cell insulin secretion and proliferation. While we observed that obese ATM EV treatment didn’t significantly change free fatty acid and triglyceride in circulation and some key mediators in adipose tissues (Figure S2F), it is possible that the ATM EVs-induced secondary effects exert profound regulation on β cell functions. In addition, it is interesting that adipsin abundance was markedly elevated in epididymal fat after treatment with obese ATM EVs (Figure S2G), suggesting the increased adipsin level as a compensatory mechanism rebalancing β cell dysfunction caused by ATM EVs [55,56].

Many EV functions have been attributed to EV-encapsulated miRNAs. Our results also have demonstrated that miRNAs are important components within obese ATM EVs. miRNA-depleted EVs derived from obese ATMs without Dicer, an essential enzyme for the synthesis of mature miRNAs, had modest effects on insulin secretion and proliferation of β cells in both in vivo and in vitro experiments. However, these studies do not exclude the potential functions of other EV components such as proteins and lipids which have been reported their profound functions in previous studies [23]. In addition, there are no reports of non-miRNA components within ATM EVs regulating their function in the context of metabolism. Previous studies have shown that the depletion of Dicer enhances proinflammatory polarization of macrophages [57,58]. We observed that obese WT and DicerKO ATMs secreted similar amount of EVs, which expressed similar levels of EV-associated markers (Figure S3C,D).

Our previous studies profiled miRNAs derived from ATM EVs in the obese state [32]. Among these miRNAs, miR-155 is highly enriched within obese ATM EVs that can be efficiently delivered into recipient cells. In addition, compared to other cell types, miR-155 is notably expressed in proinflammatory macrophages (http://biogps.org/#goto=genereport&id=100653389 accessed on 14 September 2021) [51]. Using a macrophage-depleted mouse model by clodronate liposome treatment, we further validated that macrophages are one of the main sources releasing extracellular miR-155 into β cells, as shown by much less miR-155 abundance in the islets of clodronate liposome-treated obese mice than in the controls. In addition, in vitro glucose or palmitate acid treatment repressed the miR-155 expression in islets, in contrast to the observation that miR-155 was highly expressed in obese β cells compared to the β cells isolated from lean WT mice. This suggests that a sizable component of the obesity-induced increase in miR-155 is likely derived from macrophages.

Previous studies have demonstrated the critical roles of miR-155 in various cell types, including immune cells and insulin sensitizing cells [32,51,59,60]. In the current study, we found that accumulation of miR-155 produces dual effects on β cell insulin secretion and proliferation, as shown by miR-155-mediated reduction in insulin secretion and enhancement on β cell proliferation. The addition of miR-155 inhibitor can prevent the accumulation of miR-155 as a result of obese ATM EVs incorporated into β cells, leading to the recovery of insulin secretion. A previous study by Zhu et al. has reported that depletion of miR-155 in hyperlipidemic LDL receptor knockout mouse model results in decreased percentage of insulin+ cells and plasma insulin level, suggesting the importance of miR-155 on β cell adaptation in response to hyperlipidemia [45]. Zhu et al. also show that overexpression of miR-155 increases insulin abundance [45]. However, we observed a significant reduction in the intracellular insulin content of both human and mouse islets after miR-155 accumulation. We found that the dosage of miR-155 mimics transfected into recipient cells was greatly different (2 pM versus 15 nM used in the studies by Zhu et al.), possibly resulting in this discrepancy in insulin production and secretion in in vitro experiments.

miRNAs exert their biological functions by either blocking translation and/or inducing degradation of target mRNAs by base-pairing to recognition sites [50]. Consistent with the previous report, *Mafb* is one of the target genes of miR-155 in β cells [45,61]. We found that miR-155-mediated *Mafb* repression led to increased number of proliferating ki67+ β cells in vivo and in vitro. In addition, we found that knockdown of *Mafb* can blunt insulin synthesis and glucose-stimulated insulin secretion in both human and mouse islets, accompanied by a significant repression on the expression of *Glut2* that are critical for insulin secretion. Previous studies have shown that depletion of *Mafb* in mouse pancreas impaired the development of both α cells and β cells at the postnatal stage. *Mafb* knockout still blunts α cell development and function in adulthood, whereas β cell maturation and function can be restored after 8 weeks of age [62,63]. In addition, insulin content was significantly decreased in 3 weeks old endocrine cell-specific *Mafb*^KO^ mice [62]. Although α cells are important to support β cell functions, it is interesting that Conrad et al. observed that islets isolated from *Mafb* knockout mice display comparable level of glucose-stimulated insulin secretion with WT islets [63].

In summary, our current studies have established a novel link between ATMs and β cell by secretion of miRNA-containing ATM EVs in the context of obesity. Treatment of obese ATM EVs causes a reduction in both in vivo and in vitro insulin secretion and enhanced β cell proliferation. Based on these studies, we propose that obese ATM EVs play a critical role in the endocrine signaling system which can exert profound regulation on β cell responses in the context of obesity.

## Data Availability

The datasets generated in current study are available from the corresponding authors upon reasonable request. There are no restrictions on data availability.

## Supplementary Materials

The following are available online at https://www.mdpi.com/article/10.3390/cells10092451/s1, Figure S1: The effects of obese ATM EVs on HFD WT mice, Figure S2: The effects of obese ATM EVs on metabolic phenotypes, Figure S3: Effect of obese ATM EVs on β cell apoptosis, Figure S4: Validation of dicer knockout in obese ATMs, Figure S5: Effects of miR-155 on β cell functions, Figure S6: The critical role of miR-155 in macrophage-derived EVs, Figure S7: The effect of Mafb on β cell functions.
