# Supplementary material for: Adipose Tissue Macrophages Modulate Obesity-Associated β Cell Adaptations through Secreted miRNA-Containing Extracellular Vesicles
Source: Cells. 2021 Sep 17;10(9):2451. doi: 10.3390/cells10092451 (PMC8472266; doi:10.3390/cells10092451)
Supplement: Supplementary file 1 [file cells-10-02451-s001.zip › cells-1374024-supplementary.pdf]

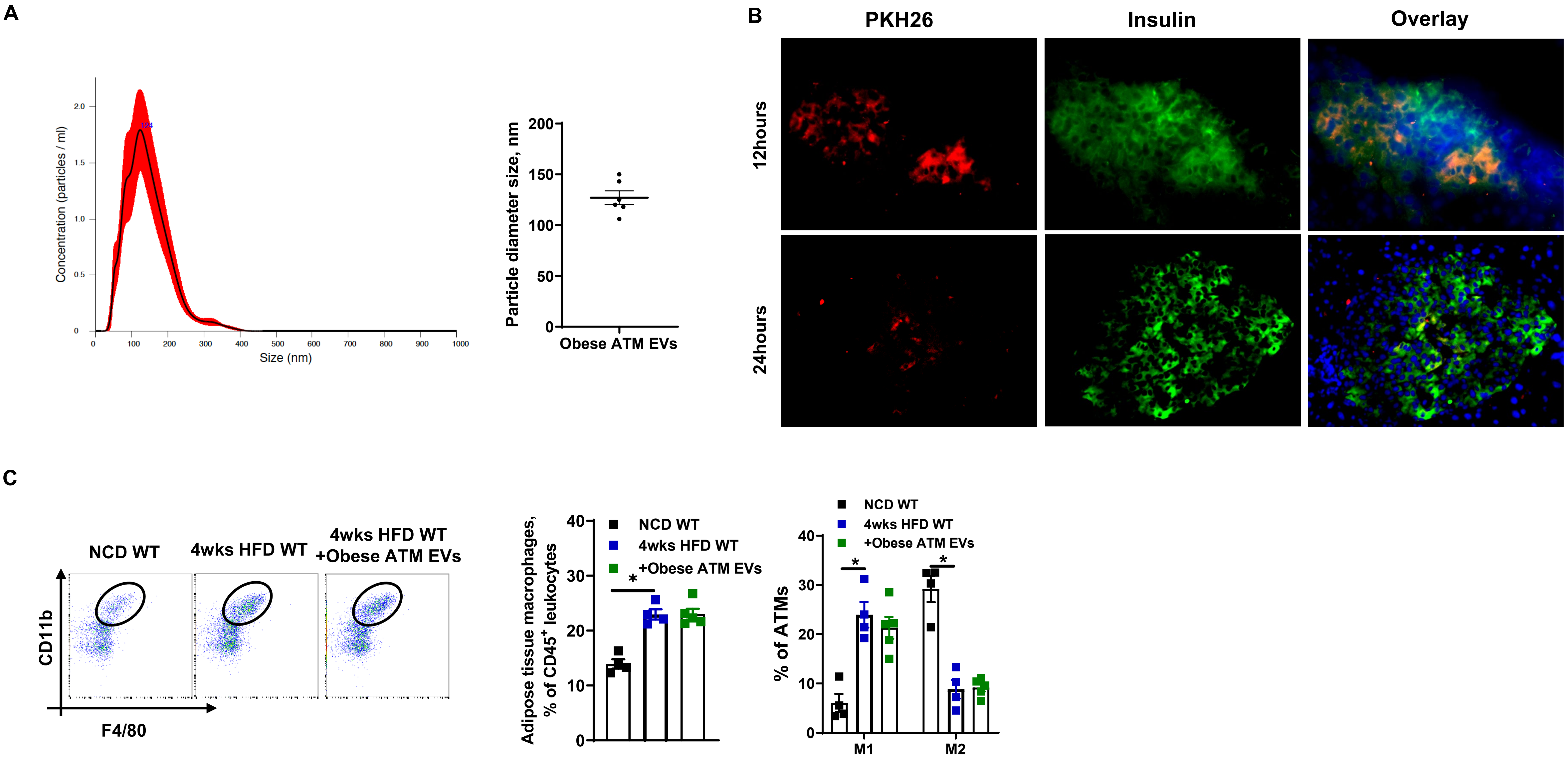

Figure S1. The effects of obese ATM EVs on HFD WT mice. (A) Particle size of obese ATM EVs analyzed by NanoSight. (B) The appearance of red fluorescence in the islets of recipients after 12 or 24 hours administration of PKH26-labeled obese ATM EVs. Representative images are shown from 3 independent experiments. (C) The population and activation of ATMs after 4 weeks treatment of obese ATM EVs. ATMs are CD45+CD11b+F4/80+ cells, and M1 and M2 ATMs were identified by CD11c+CD206- and CD11c-CD206+, respectively. Data are presented as the mean ± SEM. * P<0.05, Student's t test.

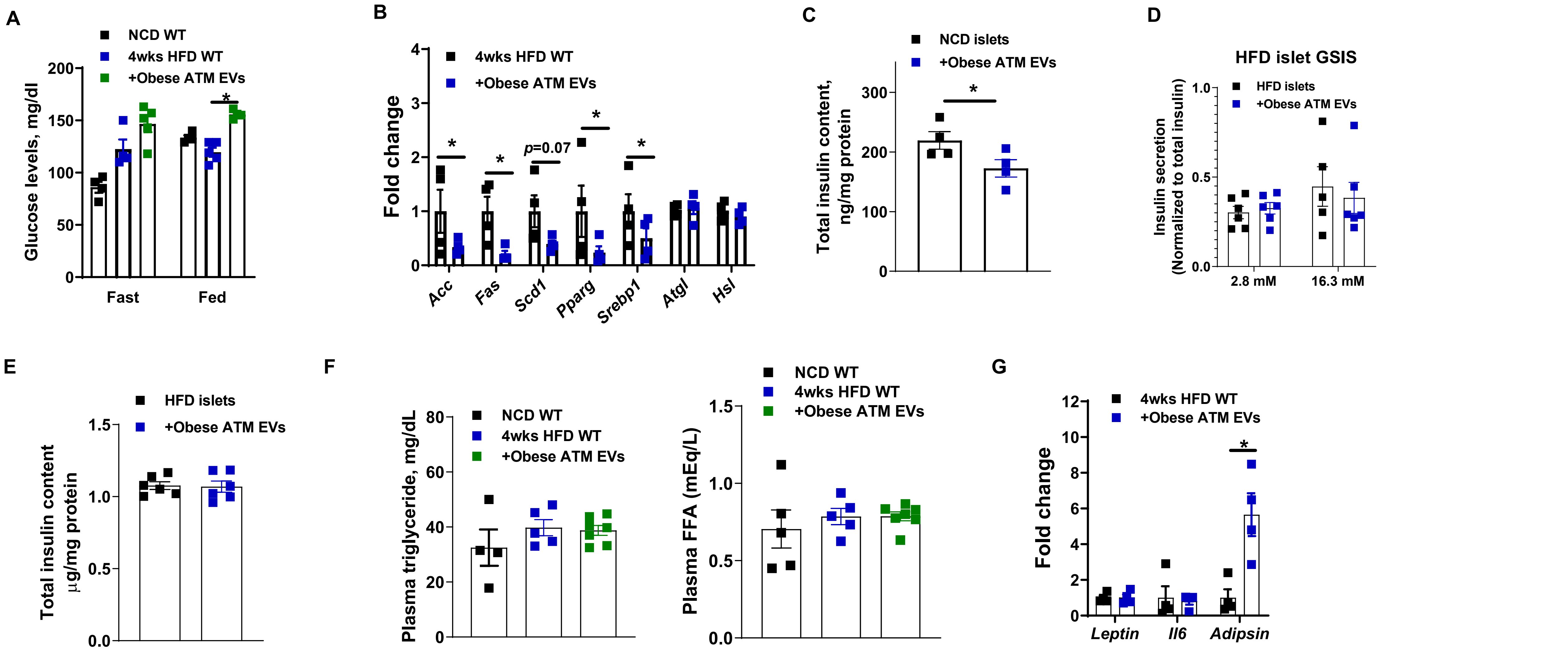

Figure S2. The effects of obese ATM EVs on metabolic phenotypes. (A) Glucose levels at either fasting or fed state after 4 weeks of obese ATM EV treatment. (B) The expression of genes associated with lipogenesis and lipolysis of epididymal fat after 4 weeks treatment of obese ATM EVs. (C) Total insulin content in NCD islets treated with obese ATM EVs. The GSIS (D) and intracellular insulin level (E) of 12wks HFD islets after treatment with obese ATM EVs. Plasma triglyceride and free fatty acid levels (F) and expression of *Leptin*, *Il6* and *Adipsin* in the epididymal fat (G) of mice treated with obese ATM EVs. Data are presented as the mean ± SEM. * *P*<0.05, Student's t test.

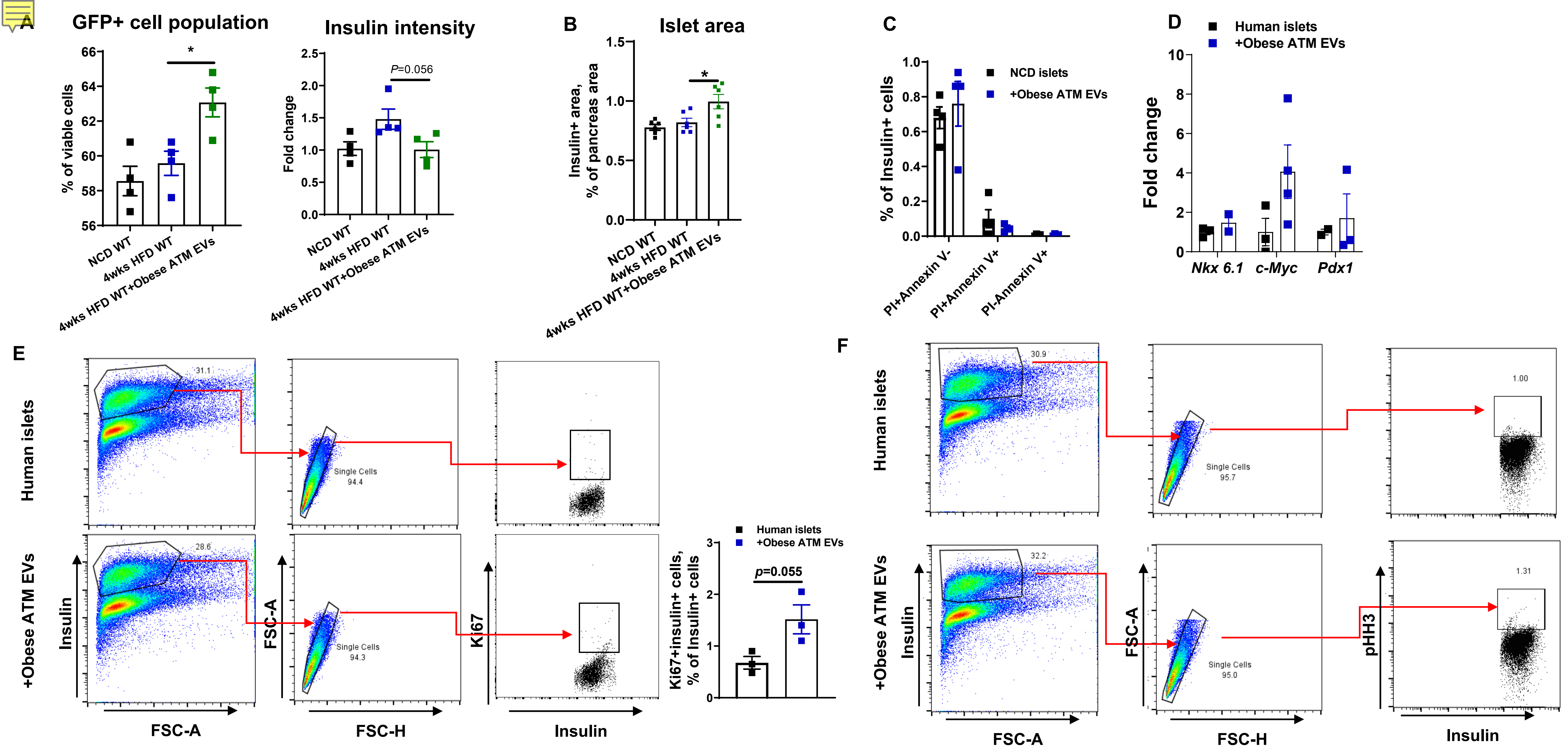

Figure S3. Effect of obese ATM EVs on β cell apoptosis. Effect of obese ATM EVs on the population of GFP+ cells and insulin intensity (A) and islet area (insulin+ cell area, B). Flow cytometric analysis was used to calculate insulin intensity by using mean fluorescent intensity of GFP signal/GFP+ cell population. The islet area data were normalized to total pancreas area. (C) Apoptosis of β cell after treatment of NCD WT islets with obese ATM EVs. (D) The abundance of genes associated with β cell proliferation in human islets after treatment with obese ATM EVs. (E and F) Flow cytometric analysis of the population of Ki67+Insulin+ or pHH3+Insulin+ cells in human islets after 72 hours treatment with obese ATM EVs. Data are presented as the mean ± SEM. * $P<0.05$, Student's t test. PI, propidium iodide. Among insulin+ cells, PI+Annexin V- cells are dead cells; PI+Annexin V+ cells are late apoptotic cells; PI-Annexin V+ cells are early apoptotic cells. FSC-A, forward scatter area; FSC-H, forward scatter height.

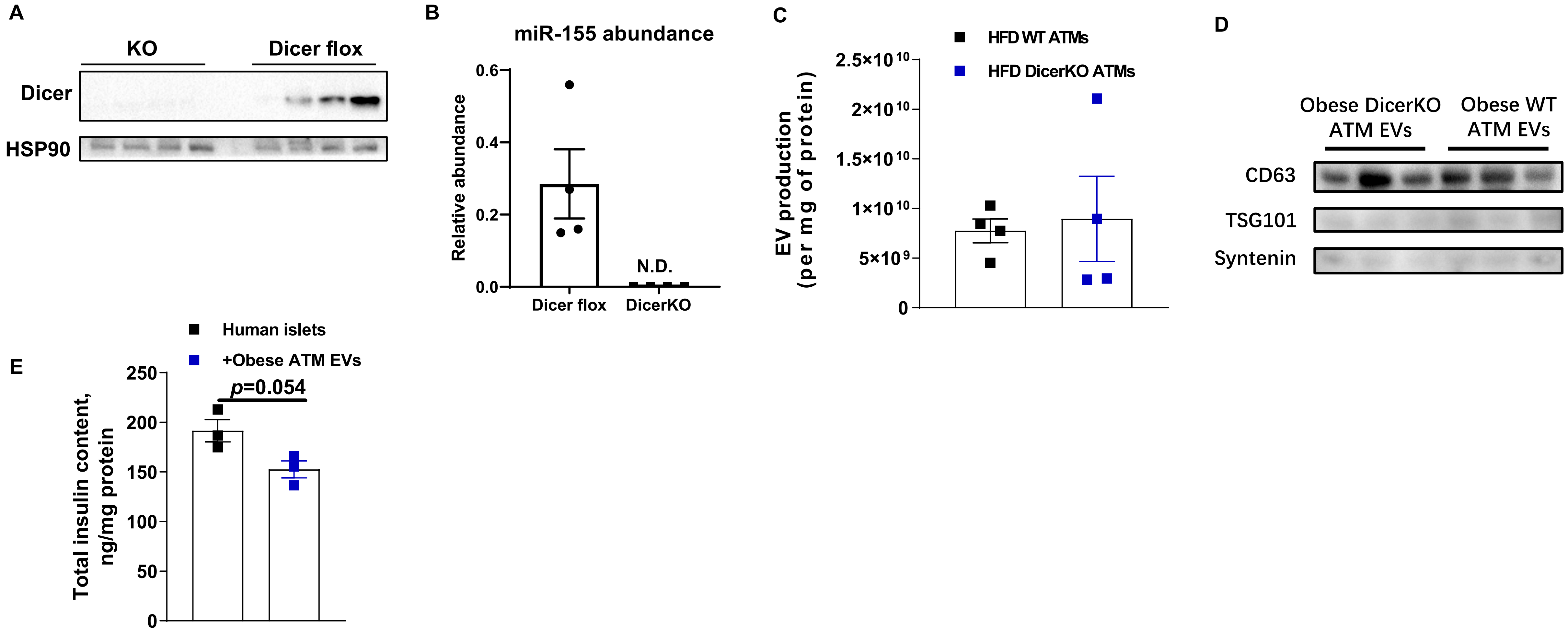

Figure S4. Validation of dicer knockout in obese ATMs. (A) The abundance of dicer in ATMs isolated from 16wks HFD-fed LysMcre-Dicer flox (KO) or Dicer flox mice. (B) miR-155 abundance in ATMs after knockout of dicer. (C) EV production from obese ATMs after knockout of Dicer. (D) The abundance of EV-associated markers of EVs derived from both obese WT and DicerKO ATMs. (E) Effect of obese ATM EVs on human islet insulin content. Data are presented as the mean ± SEM. N.D., non-detectable.

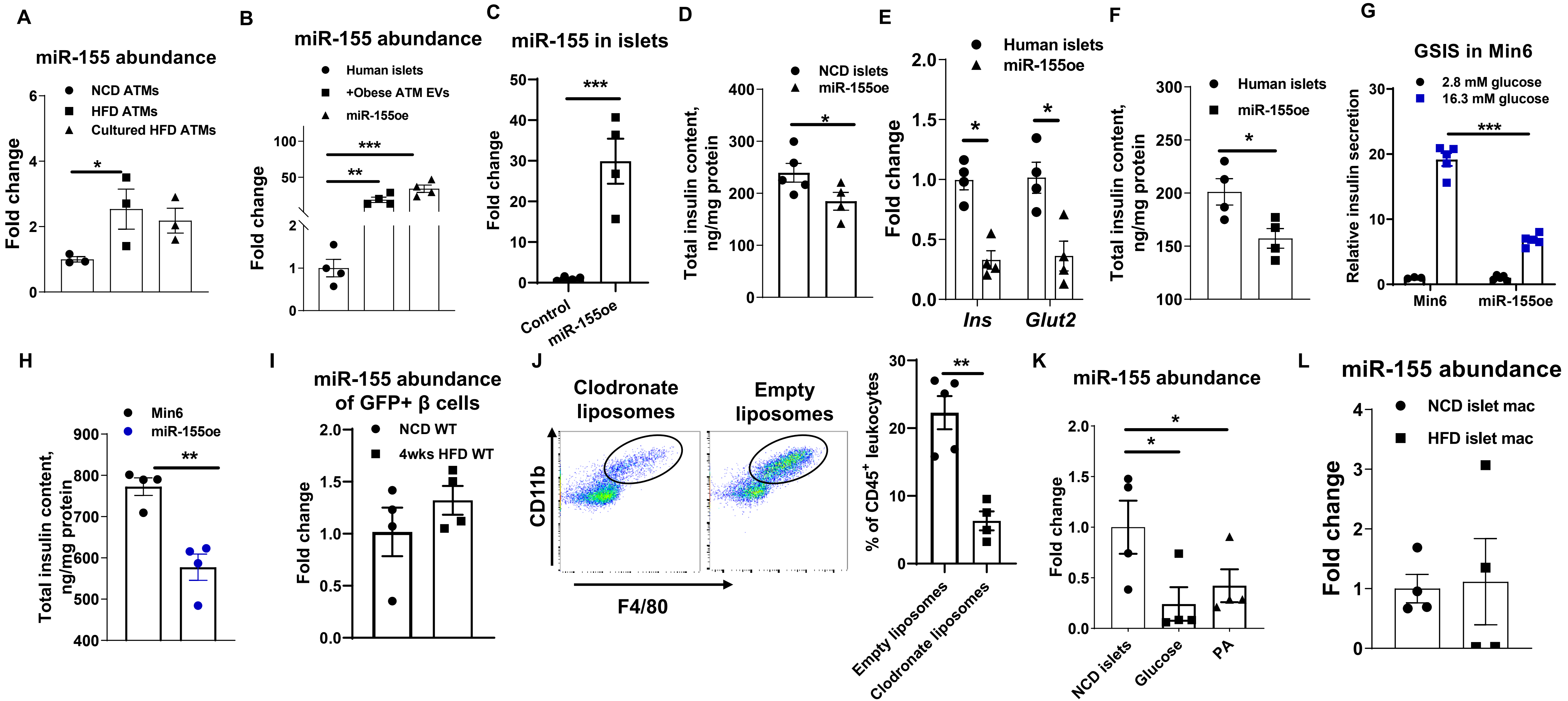

Figure S5. Effects of miR-155 on β cell functions. (A) miR-155 expression in NCD WT ATMs, 16wks HFD WT ATMs, and 24 hours cultured 16wks HFD WT ATMs. (B) miR-155 level in human islets treated with obese ATM EVs or miR-155 mimics. (C) miR-155 abundance in islets after 24 hours transfection of miR-155 mimics. (D) Total insulin content in NCD islets transfected with miR-155 mimics. Expression of Ins and Glut2 (E) and total insulin content (F) in human islets transfected with miR-155 mimics. Effects of miR-155 overexpression on GSIS (G) and intracellular insulin level (H) of Min6 cells. (I) The expression of miR-155 in GFP+ cells after 4 weeks HFD feeding. (J) The ATM population of HFD WT mice after treatment of either empty liposomes or clodronate liposomes. (K) The effect of glucose (16.3 mM) or palmitate acid (PA; 500 μM) on miR-155 levels in NCD islets. (L) miR-155 abundance of both NCD islet macrophages and 20wks HFD islet macrophages (F4/80+CD11b+). Data are presented as the mean ± SEM. * P<0.05, ** P<0.01, ** P<0.001, Student's t test.

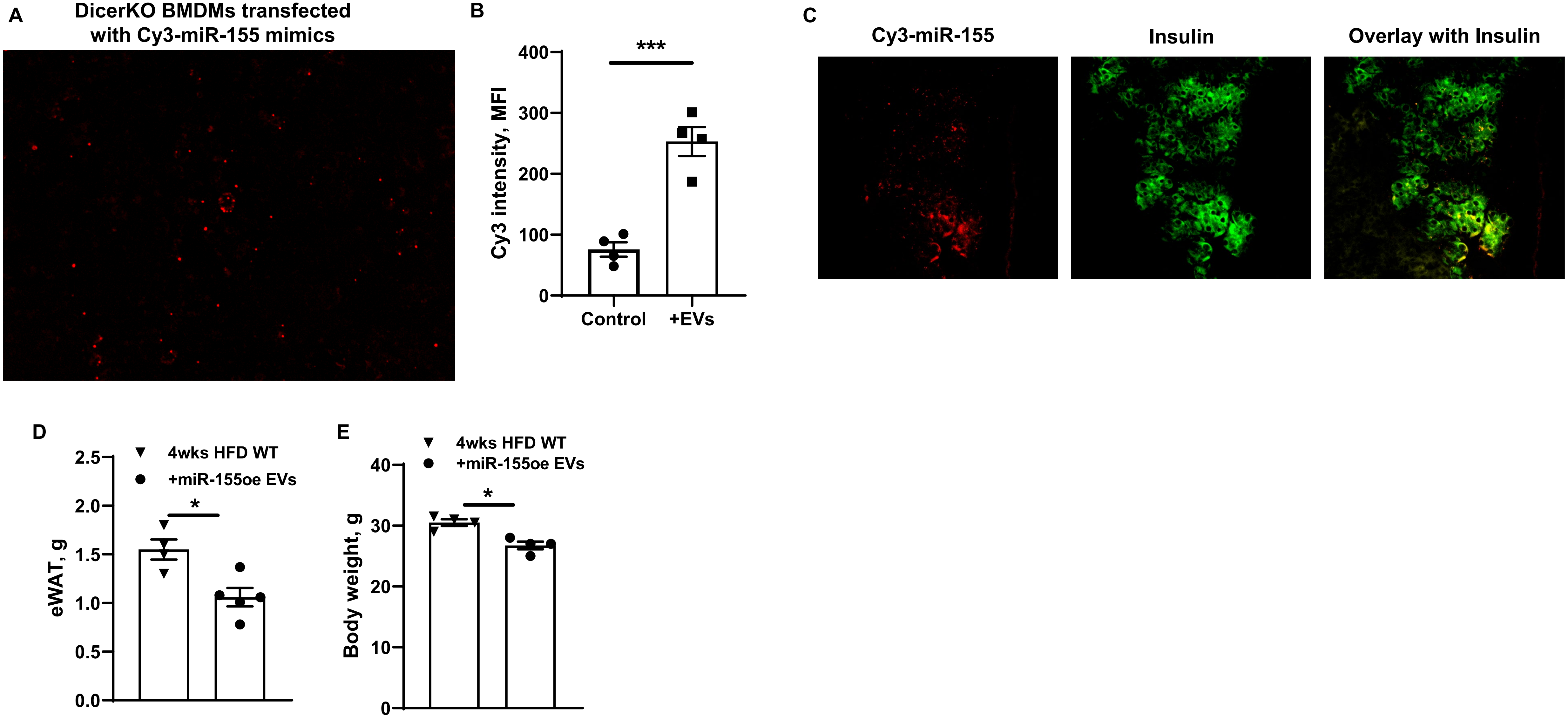

Figure S6. The critical role of miR-155 in macrophage-derived EVs. (A) The presence of Cy3 fluorescent signals in DicerKO BMDMs after 24 hours transfection of Cy3-labeled miR-155 mimics. Representative images are shown from 3 independent experiments. (B) The Cy3 intensity within BMDM-derived EVs after transfection of Cy3-miR-155 mimics. (C) The appearance of Cy3 red fluorescence in the pancreas of obese recipient mice after 24 hours injection of Cy3-miR-155 containing BMDM EVs. Representative images are shown from 3 independent experiments. (D and E) The epididymal fat mass and body weight of recipients after 4 weeks treatment of miR-155oe EVs. Data are presented as the mean ± SEM. * $P<0.05$, *** $P<0.001$, Student's t test.

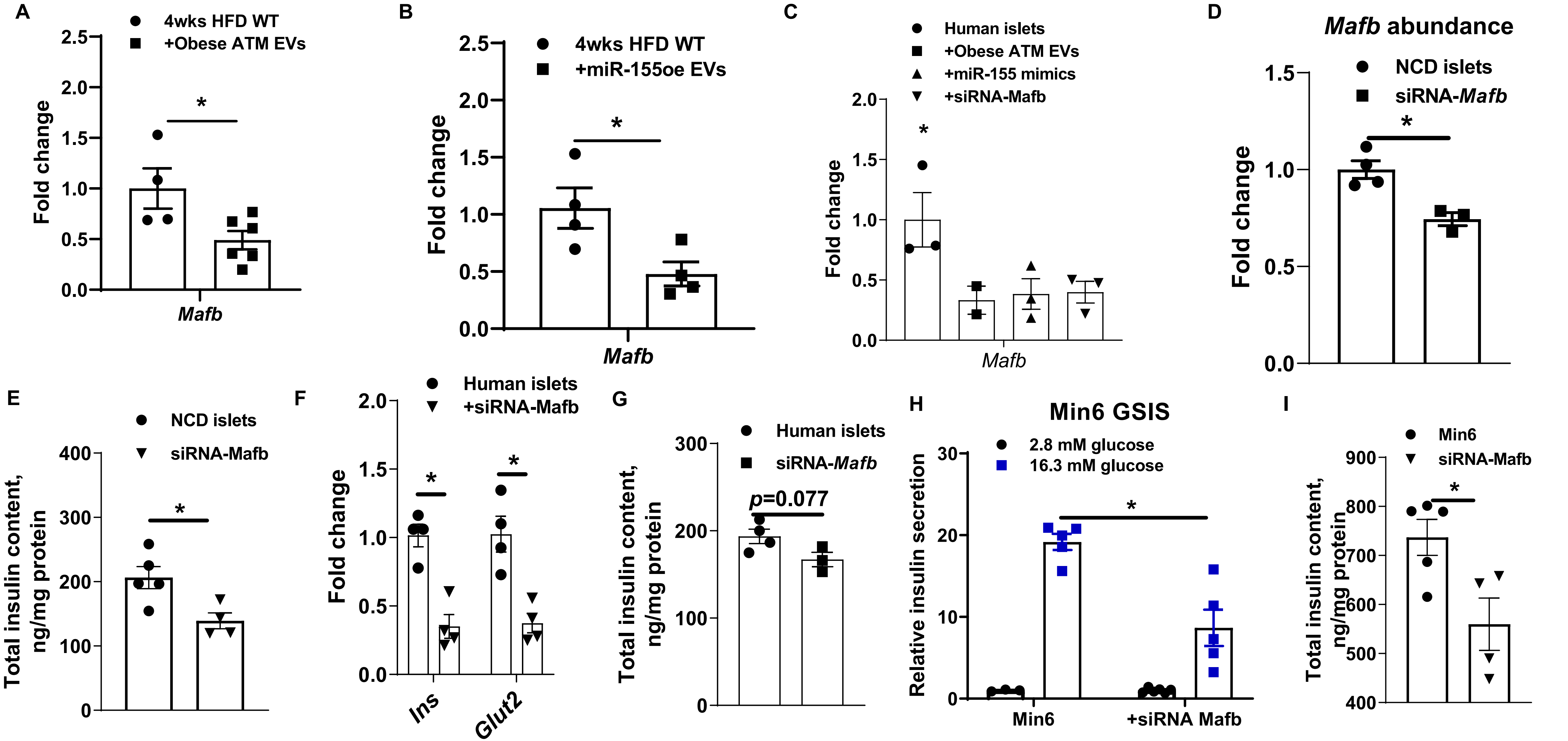

Figure S7. The effect of *Mafb* on β cell functions. (A and B) The expression of *Mafb* in GFP+ cells of recipients treated with either obese ATM EVs or miR-155oe EVs for 4 weeks. (C) *Mafb* expression in human islets after treatment with obese ATM EVs, miR-155 mimics, or siRNA-*Mafb*. (D) *Mafb* abundance in NCD islets after transfection of siRNA-*Mafb*. (E) Effect of *Mafb* knockdown on total insulin content in NCD islets. The abundance of *Ins* and *Glut2* (F) and insulin content (G) in human islets transfected with siRNA-*Mafb*. Effect of Mafb knockdown on insulin secretion (H) and total insulin level (I) in Min6 cells. Data are presented as the mean ± SEM. * $P<0.05$, Student's t test.
